# The General Movement Optimality Score-Revised (GMOS-R) with Socioeconomically Stratified Percentile Ranks

**DOI:** 10.3390/jcm13082260

**Published:** 2024-04-13

**Authors:** Christa Einspieler, Arend F. Bos, Alicia J. Spittle, Natascia Bertoncelli, Marlette Burger, Colleen Peyton, Moreno Toldo, Fabiana Utsch, Dajie Zhang, Peter B. Marschik

**Affiliations:** 1 Interdisciplinary Developmental Neuroscience—iDN, Division of Phoniatrics, Medical University of Graz, 8010 Graz, Austria; 2 Division of Neonatology, Department of Pediatrics, Beatrix Children’s Hospital, University Medical Center Groningen, University of Groningen, 9712 GZ Groningen, The Netherlands; 3 Department of Physiotherapy, Melbourne School of Health Sciences, University of Melbourne, Melbourne, VIC 3010, Australia; aspittle@unimelb.edu.au; 4 Neonatal Intensive Care Unit, Department of Medical and Surgical Sciences of Mothers, Children and Adults, University Hospital of Modena, 41124 Modena, Italy; natascia.bertoncelli@gmail.com; 5 Physiotherapy Division, Department of Health and Rehabilitation Sciences, Faculty of Medicine and Health Sciences, Stellenbosch University, Cape Town 7505, South Africa; mbu@sun.ac.za; 6 Department of Physical Therapy and Human Movement Sciences, Feinberg School of Medicine, Northwestern University, Chicago, IL 60611, USA; colleen.peyton1@northwestern.edu; 7 Department of Medical Rehabilitation, Kiran Society for Rehabilitation and Education of Children with Disabilities, Varanasi 221011, India; drmorenotoldo@gmail.com; 8 Reabilitação Infantil, Rede SARAH de Hospitais de Reabilitação, Belo Horizonte 30510-000, Brazil; fabianautsch@hotmail.com; 9 Child and Adolescent Psychiatry, Center for Psychosocial Medicine, University Hospital Heidelberg, Ruprecht-Karls University, 69115 Heidelberg, Germany; 10 Child and Adolescent Psychiatry and Psychotherapy, University Medical Center Göttingen, Leibniz-ScienceCampus Primate Cognition, 37075 Göttingen, Germany; 11 Center of Neurodevelopmental Disorders (KIND), Centre for Psychiatry Research, Department of Women’s and Children’s Health, Karolinska Institutet, 171 77 Stockholm, Sweden

**Keywords:** general movements, optimality score, preterm, sex, term, World Bank data

## Abstract

**Background:** The general movement optimality score (GMOS) quantifies the details of general movements (GMs). We recently conducted psychometric analyses of the GMOS and developed a revised scoresheet. Consequently, the GMOS-Revised (GMOS-R) instrument necessitated validation using new percentile ranks. This study aimed to provide these percentile ranks for the GMOS-R and to investigate whether sex, preterm birth, or the infant’s country of birth and residence affected the GMOS-R distribution. **Methods:** We applied the GMOS-R to an international sample of 1983 infants (32% female, 44% male, and 24% not disclosed), assessed in the extremely and very preterm period (10%), moderate (12%) and late (22%) preterm periods, at term (25%), and post-term age (31%). Data were grouped according to the World Bank’s classification into lower- and upper-middle-income countries (LMICs and UMICs; 26%) or high-income countries (HICs; 74%), respectively. **Results:** We found that sex and preterm or term birth did not affect either GM classification or the GMOS-R, but the country of residence did. A lower median GMOS-R for infants with normal or poor-repertoire GMs from LMICs and UMICs compared with HICs suggests the use of specific percentile ranks for LMICs and UMICs vs. HICs. **Conclusion:** For clinical and scientific use, we provide a freely available GMOS-R scoring sheet, with percentile ranks reflecting socioeconomic stratification.

## 1. Introduction

Since Prechtl and colleagues detected general movements (GMs) and reported their predictive validity for neurological outcomes [1,2], the translation from the scientific observation of GMs to the clinical implementation of their structured assessment has continually progressed. The Prechtl general movement assessment (GMA) [3] has increasingly benefitted from the ever-growing technical developments in both (i) data acquisition through the classic video approach (e.g., RGB and RGB-D cameras, multi-camera systems for lab recordings, and smartphone applications for home recordings) or the application of alternative sensor modalities (e.g., inertial motion sensors and pressure sensors), and (ii) data processing through artificial intelligence and machine learning approaches, with the expanding development and usage of deep learning approaches [4,5,6,7,8,9,10,11,12]. Despite this technological progress, these methods are not (yet) able to replace human clinical expertise in assessing and categorizing infants’ spontaneous movements [9]. 

The GMA is an assessment tool based on visual Gestalt perception to categorically differentiate between normal (i.e., variable sequence of neck, trunk, arm, and leg movements, waxing and waning in amplitude, speed, and intensity) and abnormal (i.e., with a lack of variability) endogenously generated motor functions, the GMs [3]. Based on the recognition of these differences and the related predictive power of certain abnormal GM patterns with respect to adverse neurological outcomes [2,3], several early specific interventions have been applied during preterm and term periods [13,14,15,16,17,18,19]. While for some interventions, there was no evidence that they altered the quality of GMs [14,16,18], others led to immediate improvement [15,17] or even the normalization [19] of GMs, although long-term benefits are not yet clear.

The change of evolving GM patterns can best be captured and quantified by a detailed assessment of the GMs [3], the general movements optimality score (GMOS) [20]. The GMOS is based on the optimality concept, introduced by Prechtl in 1980 [21], which allows us to define optimal criteria for the various movement components and quantify the scoring of the sequence, amplitude, speed, spatial range, rotations, beginning and end, and tremulousness, as well as the stiffness of GMs [20]. A higher GMOS indicates better motor performance [20,21] and is associated with a more favorable developmental outcome [22,23].

The GMOS differentiates between normal and three abnormal patterns of GMs [20,24], and the score is physiologically slightly lower after term than at preterm and term ages [20,25]. The GMOS has been shown to: (a) relate the degree of neurostructural impairments to concurrent neurofunctional representations [26,27,28]; (b) demonstrate that mechanical ventilation [29] and treatment with aminophylline [30], cerebral hypoxia [31], neonatal anemia [32], patent ductus arteriosus [29], or biliary atresia [33] impact the developing nervous system, resulting in a reduced GMOS; (c) evaluate changes or improvement due to therapeutic interventions [14,16,17,18,19]; and (d) associate the final score with neurodevelopmental outcome [22,24,28,34,35].

The first application of the GMOS to more than 780 data sets with normal and various abnormal GM patterns [20] revealed a high rate of ‘tremulous movements’ across all GM categories. The exploration of the GMOS’ psychometric properties proved the GMOS to be a reliable assessment for differentiating infants with typical outcomes from adverse outcomes (cerebral palsy and other neurodevelopmental disorders) [22]. However, a further validation study suggested omitting the item ‘tremulous movements’ and revising the scoring criteria of three other items [36]. Following these results [22,36] and based upon recent clinical evidence, we developed a new version of the GMOS, the General Movement Optimality Score–Revised (GMOS-R). The GMOS-R has a 4-point lower maximum score than the original version [20] and further refinement of other scoring criteria, thus requiring the validation and presentation of new percentile scores.

Almost 1900 GM recordings, collected in 37 countries across the 6 habitable continents and taken from very preterm age until 5^6^ weeks’ post-term age, provided the basis for the present study to address the following objectives: (i) What are the age-specific percentile ranks for the GMOS-R? (ii) How does preterm birth, sex, and whether the infant was born in a lower-middle income country (LMIC) or an upper-middle income country (UMIC), compared with a high-income country (HIC), affect the percentile ranks of the GMOS-R?

## 2. Materials and Methods

### 2.1. Comparing the GMOS-R with the GMOS

**Figure 1:**
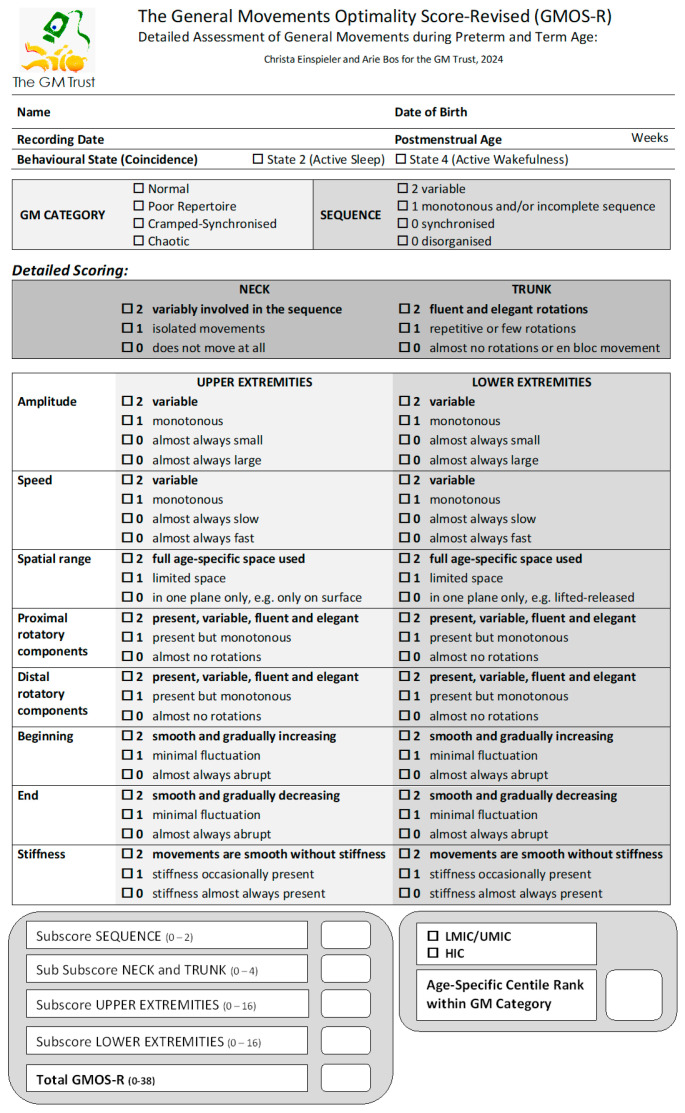
Scoresheet for the General Movements Optimality Score–Revised (GMOS-R). It is also provided in pdf format as Figure S1 in the Supplementary Materials.

The GMOS-R scoresheet is presented in Figure 1. It can be downloaded in pdf-format as Figure S1 from the Supplementary Materials. Based on the results of the validation analysis of the GMOS [22,36], the GMOS-R differs in the following ways from the original version: (a) in GMOS-R, for all items, a score of 2 refers to optimal performance, 1 refers to less optimal performance, and 0 to non-optimal performance; in GMOS, the items ‘neck’, ‘amplitude’ (for upper and lower extremities), and ‘speed’ (for upper and lower extremities) were previously scored 2 or 1 but not 0; (b) in GMOS-R, the item ‘tremulous components’ for upper and lower extremities has been removed. Additionally, we improved the description and nomenclature of specific items (examples: ‘stiffness’ instead of ‘cramped components’; ‘beginning’ instead of ‘onset’). Finally, the category ‘hypokinetic’ was removed as the GMOS-R cannot be applied if there are only isolated movements and/or startles, but no GMs are recorded during the whole observation period. 

Compared to the GMOS [20], the GMOS-R subscores for UPPER EXTREMITIES (max 16, min 0) and LOWER EXTREMITIES (max 16, min 0) changed (previously, max 18, min 2, for both subscores); NECK and TRUNK (max 4, min 0) also changed (previously, max 4, min 1), whereas the subscore for SEQUENCE (max 2, min 0) remained the same (Figure 1). As a result, the GMOS-R ranged from 0 to a maximum of 38 (optimal performance), whereas the GMOS ranged from 5 to 42 [20]. 

### 2.2. Data

We analyzed 1983 data sets of an international sample of 636 female (32.1%) and 874 male (44.1%) infants; the parents of 473 infants (23.9%) chose not to disclose the sex of their infant. The infants were videoed for their GMs because of (a) an elevated risk of neurodevelopmental disorders due to their pre-, peri-, and/or early postnatal history (81%), or (b) the parents of healthy infants volunteering to provide video recordings of their infant as a reference (19%). During a period of nearly five years (June 2018 to November 2022), neonatologists, pediatricians, pediatric neurologists, rehabilitation doctors, general practitioners, physio- and occupational therapists, community health workers, and (concerned) parents presented the video clips to the first author for a clinical evaluation of GMs. One hundred and seventy-nine (9%) data sets were also collected for other studies such as VIBeS-2 (Victorian infant brain studies) in Australia [37], prenatal exposure to COVID-19 in the USA and Brazil [38], a feasibility study to apply GMA in the USA [39], the GANESH program in rural India [40], and a pilot study to validate the intervention program MIT-PB in Spain [15].

The institutional review boards approved the recording and assessment of GMs for clinical and research purposes, and the parents consented to the clinical evaluation and publication of results. The current evaluation was approved by the Institutional Review Board of the Medical University of Graz (27-476ex14/15), Austria, and the University Medical Center, Göttingen (19/20 September 2019), Germany. 

The majority of the children (78%) were born preterm, with a median gestational age of 28 weeks (25–75th percentile 26–31; range 22–36); 10% were born at term, while for the remaining 12%, the gestational age at birth was not disclosed or not known (in vulnerable societies). Table 1 provides details of the sex assigned at birth and preterm/term birth according to the country of residence of the infants, with the majority coming from European countries (59%). 

According to the definition employed by the World Bank [41], countries of birth and residence are assigned to high-income (HICs), upper-middle-income (UMICs), and lower-middle-income countries (LMICs). In our sample, HICs comprise (alphabetically) Austria, Canada, Chile, the Czech Republic, Denmark, Germany, Israel, Italy, Japan, South Korea, Norway, Poland, Qatar, Spain, Sweden, Switzerland, the Netherlands, the UK, Uruguay, and USA (1474 video recordings; 74.3%). UMICs comprise Argentina, Azerbaijan, Brazil, China, Colombia, Kazakhstan, Mexico, Peru, South Africa, and Turkey (353 video recordings; 17.8%); LMICs comprise Bangladesh, Bolivia, Cambodia, Egypt, India, Iran, and Nepal (156 video recordings; 7.9%). The proportion of parents of healthy infants volunteering to provide video recordings of their infant was not different between LMICs and UMICs.

All infant data was recorded according to standard GMA protocol [3] between 1 and 23 weeks after birth (median 7 (25th–75th percentile 4–12)). In order to provide percentiles for each recording period, we categorized the recording age according to the postmenstrual age of the infant into the following six periods: (i) extremely preterm period (<28 weeks): *n* = 16 (0.8%); (ii) very preterm period (28^0^–31^6^ weeks): *n* = 185 (9.3%); (iii) moderate preterm period (32^0^–33^6^ weeks): *n* = 234 (11.8%); (iv) late preterm period (34^0^–36^6^ weeks): *n* = 437 (22%); (v) term period (37^0^–41^6^ weeks): *n* = 492 (24.8%); and (vi) post-term period (42^0^–45^6^ weeks): *n* = 619 (31.2%).

### 2.3. Scoring Procedure 

The scoring consisted of two steps: first, the scorer decided upon the categorical GM classification according to the common standards (Figure 1, upper part) for GM CATEGORY [3]: (i) ‘normal’ (variable sequence, amplitude, and speed, waxing and waning in intensity, and fluent and elegant due to rotations superimposed on flexion and extension); (ii) ‘poor repertoire’ (PR), i.e., the sequence, amplitude, speed, and intensity lack the normal variability; (iii) ‘cramped-synchronized’ (CS), i.e., rigid movements, where the muscles of the trunk and limbs contract almost simultaneously, thereafter relaxing almost simultaneously; and (iv) ‘chaotic’, i.e., abrupt, fast, large-amplitude movements with minimal rotations. The subscore for SEQUENCE is assigned according to the GM category: score 2 (variable) for normal, score 1 (monotonous and/or incomplete sequence) for PR GMs, and score 0 (synchronized or disorganized) for CS or chaotic GMs.

In the second step, all details (Figure 1) were scored for the neck, trunk, and upper and lower extremities by watching the video as often as necessary. This usually took five to seven replays per scoring; replaying the video at fast speed facilitated the recognition of (lack of) variability. The range of scores for each item is 0 to 2, with higher scores indicating greater optimality. Scores of 0.5 or 1.5 can be applied when necessary if a score falls between two category descriptions. In such cases, the number value of the final GMOS-R is rounded up if it is not equal to a full number. 

The GMOS-R as included in Figure 1 is freely available, and can be found in the Supplementary Materials as Figure S1. Anyone trained in GMA [3] can apply the detailed assessment process and utilize the GMOS-R; in addition, advanced training courses provided by the Prechtl General Movement Trust also focus on GMOS-R. 

### 2.4. Interscorer Agreement

Each video was scored by the first author, who is also a GM Trust tutor who regularly teaches the detailed assessment of GMs. At least one additional scorer (trained in GMOS-R and certified by the GM Trust) assessed 1368/1983 (69%) videos. The first author did not know the medical history of the infant being assessed at the time of GMOS-R scoring. Scorers were blinded to the developmental outcome. Pair-wise intra-class correlation coefficients, evaluated on the total numerical score, ranged from 0.915 to 0.993. Interrater reliability, defined as a total score difference of ≤2 points between scorers, was rated substantial to almost perfect (Cohen’s kappa values ranging between two scorers were from 0.69 to 0.98).

### 2.5. Statistics

Statistical analysis was performed using SPSS version 28.0 (SPSS Inc., Chicago, IL, USA). The GMOS-R is an ordinal scale. We provide descriptive statistics, including percentiles. Because the data were not normally distributed, the Mann–Whitney test was used to compare the GMOS-R distributions between groups (e.g., female vs. male; preterm vs. term born; LMICs and UMICs vs. HICs). The Kruskal–Wallis test was used to determine whether or not there was a significant difference between the GMOS-R distributions in infants assessed in LMICs vs. UMICs and vs. HICs. For both the Mann–Whitney test and the Kruskal–Wallis test, the presence of ties was automatically corrected by SPSS. Percentile ranks (indicated as P from now on throughout the text and in the tables) were calculated using the SPSS statistical software package. We did not smooth data to achieve exact percentiles with decimals. For example, regarding the 10th percentile, we took the cut-off for which 90% of the scores were higher, and 10% were lower. Because more infants could have these same scores, (i.e., ties), the 10th percentile is sometimes close or even similar to the minimum. A *p*-value of <0.05 (two-tailed) was considered to be statistically significant for univariate analyses; for multiple comparisons within three groups (i.e., 3 comparisons), we considered *p* < 0.017 to be significant. 

## 3. Results

The majority of GMs (*n* = 1175, 59%) were assessed as PR; 483 (25%) were scored as normal, 299 recordings (15%) as CS, and only 26 recordings (1%) were scored as chaotic GMs. In infants with normal GMs, the GMOS-R ranged from 26 to 38, with a median = 34 (25–75th percentile 32–36); infants with PR GMs had a median GMOS-R = 20 (17–24); infants with CS GMs had a median = 9 (6–11). Chaotic GMs were rare and were mainly seen in late preterm age (19/26=73%); the GMOS-R for chaotic GMs ranged from 6 to 22, with a median = 11 (8–14). 

### 3.1. Gestational Age at Birth and Sex

The GMOS-R values assessed at term age were not different between infants born preterm and infants born at term: the Mann–Whitney Z-values were −1.79 for normal GMs (*p* = 0.067), −0.99 for PR GMs (*p* = 0.322), and −0.38 for CS GMs (*p* = 0.709). Similar results were found for assessments at post-term age: Z-values were = −1.46 for normal GMs (*p* = 0.147), −1.45 for PR (*p* = 0.149), and −1.08 for CS GMs (*p* = 0.277). The GMOS-R distribution of 234 infants for whom the gestational age at birth was not disclosed did not differ from 1544 infants with a known gestational age (Z = −1.05; *p* = 0.292). 

Sex (available for *n* = 1510) did not affect the GMOS-R: Z-values were −0.82 for normal GMs (*p* = 0.414), −0.08 for PR (*p* = 0.934), and −0.95 for CS GMs (*p* = 0.343). The GMOS-R distribution for 473 infants whose sex was not disclosed was statistically not different from 1510 female or male infants (Z = −0.47; *p* = 0.637). 

### 3.2. The Infants’ Country of Birth and Residence 

All infants were born in the country where their GMs were recorded. The proportion of parents of healthy infants volunteering to provide video recordings of their infant was not different between LMICs (*n* = 110; 22%) and HICs (*n* = 265; 18%); chi-squared test, *p* = 0.26. The World Bank’s classification into LMICs and UMICs (26%) or HICs (74%) of the respective country affected the GMOS-R (Table 2). Infants with normal GMs had a lower median GMOS-R if they were born in an LMIC, compared to infants born in UMICs or HICs; also, infants from UMICs had a lower median GMOS-R than infants from HICs if their GMs were scored as normal. In other words, a GMOS-R of 35 points for a normally moving infant indicates P50 in an HIC, P75 in a UMIC, and a value above P75 in an LMIC (Table 2).

In the case of PR GMs, infants from LMICs and UMICs had lower GMOS-R medians than infants from HICs. If the GMs were scored as CS, the infants of LMICs and UMICs had a higher GMOS-R median than infants from HICs (Table 2). The GMOS-R median of infants whose GMs were scored as chaotic did not differ (Table 2). 

When only healthy infants were analyzed, the median GMOS-R of normal GMs was also lower in infants from LMICs/UMICs than from HICs (32 vs. 35, Z = −4.617; *p* < 0.001), whereas for PR GMs, no such difference was observed (GMOS-R was 21 vs. 22, Z = −1.502; *p* = 0.133).

**Figure 2:**
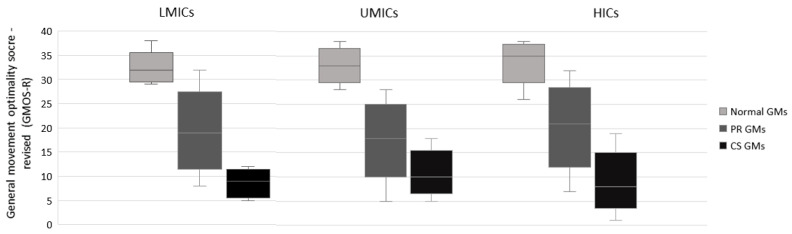
Distribution of the General Movements Optimality Score–Revised (GMOS-R) of normal GMs (light gray), PR GMs (middle gray), and CS GMs (dark gray), shown separately for LMICs, UMICs, and HICs, and presented as box-whisker plots. The boxes represent the P25–75 scores, with the horizontal line crossing the box representing the median; the whiskers represent the range of the scores. Key: CS, cramped-synchronized; PR, poor repertoire.

Figure 2 presents graphically the GMOS-R for normal GMs, PR GMs, and CS GMs. Notably, the GMOS-R of normal GMs in the lower range overlaps with those of PR GMs in the higher range. Similarly, the GMOS-R of PR GMs in the lower range overlaps with those of CS GMs in the higher range. No overlap occurs between normal GMs and CS GMs (Figure 2).

### 3.3. Distribution of GMOS-R with Recording Age-Specific Percentiles

In Table 3, we present the distribution of normal and abnormal GM patterns according to the different recording age groups, shown separately for LMICs, UMICs, and HICs. To calculate percentile ranks, we combined the results of LMICs and UMICs because the numbers of recordings in those two categories were rather small, and the differences between those two were the smallest.

Based on these subsamples and the results presented in Table 2, we provide GMOS-R percentile ranks for each age group with a minimum of 15 infants assessed for the preterm period, term period, and post-term period separately for LMICs/UMICs and HICs (Table 4). It provides a concise and precise description of the experimental results and their interpretation, as well as the basis for the experimental conclusions that can be drawn. We then repeated the analyses to assess differences between infants from LMICs/UMICs vs. HICs separately for the late preterm, term, and post-term periods. These results were consistent with those reported for the complete period (Table 2), with the exception of CS GMs; in the term and post-term periods, the difference between birth country was not statistically significant in infants with CS GMs. 

## 4. Discussion

In this study, we provide the new age-specific GMOS-R for scientific and clinical use, including recording age-specific percentile ranks separately for the infants’ country of residence for LMICs and UMICs and for HICs. The GMOS-R did not differ between sexes, nor between preterm and term infants when assessed at term or post-term. The GMOS-R in infants from LMICs and UMICs was lower than in infants from HICs. 

More than 30 years ago, a detailed assessment for preterm- and term-age GMs was introduced [42] that proved useful for showing the associations between GMs and dose-dependent maternal [43] or infant medications [44]. In 2016, we introduced separate scoring for movements in the upper and lower extremities after we had empirical proof that, for example, stiffness occurred more frequently in the legs than in the arms [20]. The recent results of the Rasch analysis of the GMOS [22,36] led to the current report presenting a revised protocol, together with new percentile ranks. At the same time, we aimed to explore whether the detailed GMA of infants from around the world presents similar findings or significant differences. Since more than 80% of preterm births worldwide occur in Asia and sub-Saharan Africa [45], it is essential to report data from LMICs, UMICs, and HICs as lower- and middle-income countries are often underrepresented in the literature. Every year, around 1500 trainees worldwide are certified by the GM Trust in LMICs [19], UMICs [23,25,28,35], and HICs [14,16,24,26,27,29,30,31,32,33,34]. 

Although our sample is a convenience sample, it is representative of infants assigned to GMA because of their medical history, clinical urgencies, and/or parental concerns. Hence, in accordance with previous observations [46,47], PR GMs were the most frequently observed movement pattern (59% of all recordings), which increased when the infant was recorded before 34 weeks’ postmenstrual age to 69–77%. Interestingly, GMOS-R distributions were affected by the country’s gross national income. For example, a GMOS-R of 21 in an infant with PR GMs is on P50 in an HIC, but between P50 and P75 in LMICs and UMICs. Differences are also dependent on the infant’s postmenstrual age when recording. At late preterm, term, or post-term age, the median difference in infants with PR GMs is only 1 to 2 points lower in LMICs and UMICs than in HICs, but the difference is much more substantial in infants recorded at younger than 32 weeks. A GMOS-R of 14, for example, indicates P50 in an infant with PR GMs in LMICs and UMICs, but below P10 if this infant is born and lives in an HIC. 

It is particularly noteworthy that the World Bank’s classification into LMICs and UMICs of the infant’s country of residence also plays a role regarding normal GMs. For example, a GMOS-R of 35 is above P75 in an LMIC, on P75 in an UMIC, and on P50 at an HIC. Apparently, less optimal normal GMs occur more often in infants from LMICs and UMICs, which result in percentile ranks of above P25 in these countries, whereas the less optimal normal GMs are below P25 in HICs. 

We have thought of several possible explanations for these differences between countries of birth and residence. Apart from the unequal sample size, with this sample being lower in LMICs and UIMCs than in HICs, several factors may have contributed to these different GMOS-R scores. The neonatal and early postnatal disease burden in UMICs and LMICs is higher than in HICs, for example, due to a greater incidence of severe infections and meningitis, asphyxia, and severe intracranial hemorrhages. It could, therefore, be that we included more severe cases from LMICs and UMICs than from HICs. However, the fact that we confirmed the lower GMOS-R in LMICs/UMICs compared with HICs when analyzing only healthy infants indicates that other factors are important. These factors are related to socio-economic circumstances; mother–infant dyads residing in lower-resource countries are disproportionally affected by multiple socioeconomic and environmental risk factors, such as poverty, less education, high unemployment levels, exposure to violence in the community and at home, inadequate nutrition, and health care that may compromise the mother’s health and her child’s development with less access to therapy [48]. As a consequence, factors that may be associated with the somewhat lower GMOS-R include poor coverage of essential antenatal care, adolescent pregnancy, malaria and HIV infections during pregnancy, urine and cervical infections during pregnancy, the undernutrition of pregnant women, home deliveries with limited access to skilled delivery, and less optimal neonatal management (a lack of available resources and trained staff) [49,50,51]. Taken together, this may have led to the overall lower GMOS-R that we found within both normal GMs and PR GMs in UMICs and LMICs. We need to collect more reference data from middle-income countries because, currently, recordings are unbalanced in their number. More importantly, we urgently need to shed more light on which factors particularly affect GMOS-R in LMICs and UMICs to find appropriate ways to improve them. In this respect, it is hopeful that a significant reduction in mortality rates has been reached for infants born preterm over the past three decades in LMICs and UMICs [52].

As described previously [20], CS GMs, which are early markers for spastic cerebral palsy [2,53], hardly occurred (2%) before moderate preterm age. Although the GMOS-R of infants with CS GMs was also significantly affected by the economic condition of the infants’ country of residence, this difference was less pronounced regarding the various recording ages. This could be explained by there being fewer recordings of CS GMs from UMICs before term age (actually, none from LMICs). Chaotic GMs were also rare in our sample (1%) and occurred—as described previously [3,20]—mainly at late preterm age. 

Because GMs are innate motor behaviors [1,3], it has not been found that sex affects their presence and appearance [54]. Even though there is growing evidence of sex effects in evolving neurodevelopmental conditions [55], we did not find any sex effects related to GMs in our large sample. Of note, the percentage of male infants in our sample was higher than that of female infants. Because nearly 80% of our sample comprised infants considered to be at risk for developmental problems, this may be a reflection that male sex is associated with an increased rate of risk factors for abnormal neurodevelopment. However, in case of increased risk, the GMOS-R was not different between males and females. 

We could not demonstrate that infants born preterm differ in their GMOS-R from infants born at term when it was recorded during the term or post-term periods. This was unexpected, as a meticulous description—though published more than 30 years ago—of term-equivalent GMs in preterm-born infants revealed fewer rotations compared to their term-born peers [56]. The reason for this might not only be improved neonatal management but also that the sample with normal GMs studied here at term and post-term age is more than 5 times larger (*n* = 186 from HICs) than the term-born reference sample (*n* = 37 from HICs) [56]. 

### 4.1. Limitations 

We recognize several limitations to this study. First, this dataset is a sample of convenience, with many cases submitted for a second opinion. The sample is not reflective of the population, with more ‘at-risk’ infants being included; therefore, it is more likely to include children with abnormal GMs and lower GMOS-R scores. Despite this potential limitation, the range of GMOS-R scores differs by GM categories, adding further validity to the scale. For example, the median GMOS-R of 34 for all the recordings assessed as normal indicates that 50% of the 483 infants with normal GMs scored in the optimal range (highest 10%, i.e., a GMOS-R of 34 to 38). In contrast, GMOS-R values are only between 1 and 5 if infants had a CS GM score at or below P10. 

Second, preterm-born infants are overrepresented in our sample. The clinical application of GMA, including the GMOS-R, still focuses mainly on preterm-born infants, and studies with a detailed assessment of GMs in infants born at term are scarce [34,53,55]. Despite the inclusion of 205 term-born infants in our sample, it was not the aim of this study to analyze the GMOS-R distribution in infants with specific conditions, such as, for example, hypoxic-ischaemic encephalopathy or congenital anomalies. Regardless of the infant’s underlying condition, the provision of percentile ranks across a wide range of postmenstrual ages in LMICs and UMICs and in HICs can assist clinicians and researchers in understanding the infant’s GMOS-R score relative to their peers, as categorized by the location of birth. 

Third, infants from HICs are overrepresented, making up 74% of our sample. HICs account for a minor proportion of the world’s population [57] and for only an estimated 10% of all preterm births worldwide [58]. Although GMA has been taught since 1997 [2], it was roughly 15 years later that it gained a foothold in LMICs and UMICs. The GMA instrument may be of even greater diagnostic value in these countries because it is cost-efficient and its predictive values are not inferior, and are perhaps even superior, to expensive neuroimaging tools, which are not as readily available in LMICs and UMICs [59,60].

Finally, it was beyond the scope of this study to associate GMOS-R percentile ranks with the later developmental outcome of a child. This important question needs to be addressed in further studies including this sample when the child’s age allows for a reliable diagnosis or outcome reporting. 

### 4.2. A Note of Caution

Detailed assessment is more time-consuming than the Gestalt perception and classification of normal or abnormal GM categories. Focusing on the details of movements (Figure 1) also interferes with the perception of the GM Gestalt. Hence, an assessment should always start with the categorical GM scoring, followed by the detailed GMOS-R assessment.

Finally, we need to be aware of a certain overlap within the scores. For example, the maximum GMOS-R of PR GMs (i.e., 32) corresponds to P50 (in LMICs) and to scores below P25 (in HICs) of normal GMs. This demonstrates the need to interpret the GMOS-R within the context of the GM categories. It is also important to keep in mind that a difference in GMOS-R of 1 or 2 points between observers is not clinically relevant. More importantly, a particular GMOS-R should be considered in relation to its percentile, which gives more information on the status of the infant’s neurological condition than the categorical classification alone. 

## 5. Conclusions

The GMA is an important tool to assess the neurological status and well-being of an infant. It has proven useful in clinical practice and research over the last 30 years. The detailed scoring of neuromotor functions beyond the overall Gestalt appearance bears the power to decipher the amelioration or deterioration of an infant’s neurological condition and find associations with potential perinatal and neonatal risk factors. In addition, with the ever-growing undertakings to study early intervention strategies, starting in the NICU soon after birth, the GMOS-R has the potential to document subtle changes and evaluate immediate therapeutic success. As a complementary tool to the categorical GMA, the GMOS-R (Figure 1) provides a dimensional concept, which we suggest applying in research and clinical settings for documenting the precise differences between groups or describing developmental changes and therapeutic success.

## Figures and Tables

**Table 1:** Country of residence, sex assigned at birth, and preterm vs. full-term birth of 1983 infants assessed with the GMOS-R scoresheet.

	Female/Male	Pre-/Fullterm	Total
North America			
UMICs ^a^ (*n* = 10)	2/4	5/4	
HICs ^b^ (*n* = 120)	24/32	78/29	130 (6.6%)
Not diclosed/not known	68	14	
South America			
LMICs ^c^ and UMICs ^d^ (*n* = 123)	44/46	85/5	
HICs ^e^ (*n* = 18)	7/11	16/2	141 (7.1%)
Not diclosed/not known	33	33	
Europe			
HICs ^f^	429/625	1023/50	1168 (58.9%)
Not diclosed/not known	114	95	
Africa			
LMICs ^g^ and UMICs ^h^	39/38	112/21	151 (7.6%)
Not diclosed/not known	74	18	
Asia			
LMICs ^i^ and UMICs ^j^ (*n* = 225)	58/64	99/80	
HICs ^k^ (*n* = 65)	11/19	46/9	290 (14.6%)
Not diclosed/not known	138	56	
Australia and NewZealand			
HICs	22/35	46/9	103 (5.2%)
Not disclosed/not known	46	56	
Total	636/874	1544/205	1983 (100%)
Not disclosed/not known	473	234	

^a^ Mexico; ^b^ Canada, USA; ^c^ Bolivia; ^d^ Argentina, Brazil, Colombia, Peru; ^e^ Chile, Uruguay; ^f^ Austria, Czech Republic, Denmark, Germany, Italy, Norway, Poland, Spain, Sweden, Switzerland, The Netherlands, UK; ^g^ Egypt; ^h^ South Africa; ^i^ Bangladesh, Cambodia, India, Iran, Nepal; ^j^ Azerbaijan, China, Kazakhstan, Turkey; ^k^ Israel, Japan, Korea, Qatar [41].

**Table 2:** GMOS-R percentile ranks, according to normal and abnormal general movement (GM) patterns for infants recorded in lower-middle-income countries (LMICs, *n* = 156), upper-middle-income countries (UMICs, *n* = 353), and high-income countries (HICs, *n* = 1474) [41].

	LMICs	UMICs	HICs
	Median = 32	Median = 33	Median = 33
	P25–P75 = 30–33	P25–P75 = 31–35	P25–P75 = 33–37
Normal GMs	Min–Max = 29–38	Min–Max = 28–38	Min–Max = 26–38
	*n* = 50	*n* = 78	*n* = 355
	**LMICs vs. UMICs: Z = −2.61; *p* = 0.009**		
	**LMICs vs. HICs: Z = −5.58; *p* < 0.001**		
	**UMICs vs. HICs: Z = −3.59; *p* < 0.001**		
	**LMICs and UMICs vs. HICs: Z = −5.84; *p* < 0.001**		
	Median = 19	Median = 18	Median = 21
	P25–P75 = 15–23	P25–P75 = 15–22	P25–P75 = 17–25
PR GMs	Min–Max = 8–32	Min–Max = 5–28	Min–Max = 7–32
	*n* = 91	*n* = 236	*n* = 848
	LMICs vs. UMICs: Z = 1.14; *p* = 0.253		
	**LMICs vs. HICs: Z = −3.34; *p* < 0.001**		
	**UMICs vs. HICs: Z = −6.85; *p* < 0.001**		
	**LMICs and UMICs vs. HICs: Z = −7.16; *p* < 0.001**		
	Median = 9	Median = 10	Median = 8
	P25–P75 = 6–11	P25–P75 = 8–13	P25–P75 = 6–11
CS GMs	Min–Max = 5–12	Min–Max = 5–18	Min–Max = 1–19
	*n* = 13	*n* = 35	*n* = 251
	LMICs vs. UMICs: Z = −1.63; *p* = 0.102		
	LMICs vs. HICs: Z = 0.34; *p* = 0.735		
	**UMICs vs. HICs: Z = 3.16; *p* = 0.002**		
	**LMICs and UMICs vs. HICs: Z = 2.80; *p* = 0.005**		
			Median = 11
			P25–P75 = 8–15
Chaotic GMs	Min–Max = 11–16	Min–Max = 8–14	Min–Max = 6–22
	*n* = 2	*n* = 4	*n* = 20
	LMICs and UMICs vs. HICs: Z = −0.31; *p* = 0.760		

Mann–Whitney test. Key: CS, cramped-synchronized; P, percentile rank; PR, poor repertoire. Bold values indicate statistically significant differences, corrected for multiple comparisons.

**Table 3:** Number of normal and abnormal general movement (GM) patterns according to the age of assessment and the infants’ country of birth and residence.

Age Period of Assessment	Normal GMs	PR GMs	CS GMs	Chaotic GMs	Total
Extremely preterm					*n* = 16
LMICs	0	0	0	0	
UMICs	0	8	0	0	8
HICs	5	3	0	0	8
Distribution	31%	69%			
Very preterm					*n* = 185
LMICs	2	4	0	0	6
UMICs	1	5	1	0	7
HICs	35	134	3	0	172
Distribution	21%	77%	2%		
Moderate preterm					*n* = 234
LMICs	1	3	0	0	4
UMICs	3	9	0	0	12
HICs	35	150	32	1	218
Distribution	17%	69%	14%		
Late preterm					*n* = 437
LMICs	6	10	0	1	17
UMICs	14	29	7	2	52
HICs	94	180	78	16	368
Distribution	26%	50%	20%	4%	
Term					*n* = 492
LMICs	9	26	6	1	42
UMICs	28	76	16	2	122
HICs	71	176	78	3	328
Distribution	22%	57%	20%	1%	
Post-term					*n* = 619
LMICs	32	48	7	0	87
UMICs	32	109	11	0	152
HICs	115	205	60	0	380
Distribution	29%	58%	13%		
Total					*n* = 1983
LMICs	50	91	13	2	156
UMICs	78	236	35	4	353
HICs	355	848	251	20	1474

Key: CS, cramped-synchronized; PR, poor repertoire.

**Table 4:** GMOS-R percentile ranks according to the different general movement (GM) patterns assessed at the very preterm period (including 16 infants recorded at <28 weeks’ gestation), moderate preterm, late preterm, term, and post-term periods for LMICs and UMICs and for HICs [41], with a sample size of at least *n* = 15 per group.

**Recording Age**		**<31^6^ Weeks**				**32^0^–33^6^ Weeks**					**34^0^–36^6^ Weeks**				
		**N**		**PR**		**N**		**PR**	**CS**		**N**		**PR**		**CS**
		*n* = 3		*n* = 17		*n* = 4		*n* = 12	*n* = 0		*n* = 20		*n* = 39		*n* = 7
**LMICs and UMICs**	Max	n.a.		24		n.a.		n.a.	n.a.		37		27		n.a.
	P90			23							37		25		
	P75			19							34		22		
	**P50**			**14**							**32**		**20**		
	P25			12							31		15		
	P10			8							29		10		
	Min			8							29		5		
			
**Recording Age**		**<31^6^ Weeks**				**32^0^–33^6^ Weeks**					**34^0^–36^6^ Weeks**				
		**N**		**PR**		**N**		**PR**	**CS**		**N**		**PR**		**CS**
		*n* = 40		*n* = 137		*n* = 35		*n* = 150	*n* = 32		*n* = 94		*n* = 180		*n* = 78
**HICs**	Max	38		32		38		32	16		38		32		18
	P90	37		31		38		31	15		38		27		13
	P75	35		28		37		28	12		37		25		11
	**P50**	**34**		**23**		**35**		**23**	**8**		**36**		**21**		**7**
	P25	32		18		33		19	6		33		17		5
	P10	31		15		31		16	3		32		14		4
	Min	26		9		31		7	2		29		8		1
		
**Recording Age**			**37^0^–41^6^ Weeks**							**42^0^–45^6^ Weeks**					
			**N**		**PR**		**CS**			**N**		**PR**		**CS**	
			*n* = 37		*n* = 102		*n* = 22			*n* = 64		*n* = 157		*n* = 18	
**LMICs and UMICs**	Max		38		28		17			38		32		17	
	P90		37		26		13			36		25		14	
	P75		34		23		12			35		22		11	
	**P50**		**33**		**18**		**10**			**33**		**19**		**9**	
	P25		31		15		7			31		16		8	
	P10		30		11		6			29		14		5	
	Min		29		6		5			28		10		5	
		
**Recording Age**			**37^0^–41^6^ Weeks**							**42^0^–45^6^ Weeks**					
			**N**		**PR**		**CS**			**N**		**PR**		**CS**	
			*n* = 71		*n* = 176		*n* = 78			*n* = 115		*n* = 204		*n* = 60	
**HICs**	Max		38		32		19			38		32		16	
	P90		38		28		14			37		27		15	
	P75		37		24		11			36		24		12	
	**P50**		**35**		**20**		**9**			**34**		**20**		**9**	
	P25		31		17		6			32		16		6	
	P10		28		14		4			30		14		5	
	Min		26		8		3			26		7		1	

Key: CS, cramped-synchronized; HICs, high-income countries; LMICs, lower-middle-income countries; N, normal; P, percentile rank; PR, poor repertoire; UMICs, upper-middle-income countries.

## Data Availability

The data presented in this study are available on request from the corresponding author. The data are not publicly available due to the sensitivity of the pseudonymized data and because the participant consent for the collection of data did not explicitly or implicitly include sharing their data at a public repository.

## Supplementary Materials

The following supporting information can be downloaded at: https://www.mdpi.com/article/10.3390/jcm13082260/s1, Figure S1: the GMOS-R scoresheet.
