# Supplementary material for: The General Movement Optimality Score-Revised (GMOS-R) with Socioeconomically Stratified Percentile Ranks
Source: J Clin Med. 2024 Apr 13;13(8):2260. doi: 10.3390/jcm13082260 (PMC11050782; doi:10.3390/jcm13082260)
Supplement: Supplementary file 1 [file jcm-13-02260-s001.zip › jcm-2858770-supplementary.pdf]

The General Movements Optimality Score-Revised (GMOS-R)

Detailed Assessment of General Movements during Preterm and Term Age:

Christa Einspieler and Arie Bos for the GM Trust, 2024

The GM Trust

**Name**		**Date of Birth**	
**Recording Date**		**Postmenstrual Age**	Weeks
**Behavioural State (Coincidence)**	☐ State 2 (Active Sleep)	☐ State 4 (Active Wakefulness)	

**GM CATEGORY**	☐ Normal<br>☐ Poor Repertoire<br>☐ Cramped-Synchronised<br>☐ Chaotic	**SEQUENCE**	☐ 2 variable<br>☐ 1 monotonous and/or incomplete sequence<br>☐ 0 synchronised<br>☐ 0 disorganised

Detailed Scoring:

NECK	TRUNK
☐ **2 variably involved in the sequence**	☐ **2 fluent and elegant rotations**
☐ **1** isolated movements	☐ **1** repetitive or few rotations
☐ **0** does not move at all	☐ **0** almost no rotations or en bloc movement

	UPPER EXTREMITIES	LOWER EXTREMITIES
**Amplitude**	☐ **2 variable**	☐ **2 variable**
	☐ **1** monotonous	☐ **1** monotonous
	☐ **0** almost always small	☐ **0** almost always small
	☐ **0** almost always large	☐ **0** almost always large
**Speed**	☐ **2 variable**	☐ **2 variable**
	☐ **1** monotonous	☐ **1** monotonous
	☐ **0** almost always slow	☐ **0** almost always slow
	☐ **0** almost always fast	☐ **0** almost always fast
**Spatial range**	☐ **2 full age-specific space used**	☐ **2 full age-specific space used**
	☐ **1** limited space	☐ **1** limited space
	☐ **0** in one plane only, e.g. only on surface	☐ **0** in one plane only, e.g. lifted-released
**Proximal rotatory components**	☐ **2 present, variable, fluent and elegant**	☐ **2 present, variable, fluent and elegant**
	☐ **1** present but monotonous	☐ **1** present but monotonous
	☐ **0** almost no rotations	☐ **0** almost no rotations
**Distal rotatory components**	☐ **2 present, variable, fluent and elegant**	☐ **2 present, variable, fluent and elegant**
	☐ **1** present but monotonous	☐ **1** present but monotonous
	☐ **0** almost no rotations	☐ **0** almost no rotations
**Beginning**	☐ **2 smooth and gradually increasing**	☐ **2 smooth and gradually increasing**
	☐ **1** minimal fluctuation	☐ **1** minimal fluctuation
	☐ **0** almost always abrupt	☐ **0** almost always abrupt
**End**	☐ **2 smooth and gradually decreasing**	☐ **2 smooth and gradually decreasing**
	☐ **1** minimal fluctuation	☐ **1** minimal fluctuation
	☐ **0** almost always abrupt	☐ **0** almost always abrupt
**Stiffness**	☐ **2 movements are smooth without stiffness**	☐ **2 movements are smooth without stiffness**
	☐ **1** stiffness occasionally present	☐ **1** stiffness occasionally present
	☐ **0** stiffness almost always present	☐ **0** stiffness almost always present

Subscore SEQUENCE (0 – 2)	
Sub Subscore NECK and TRUNK (0 – 4)	
Subscore UPPER EXTREMITIES (0 – 16)	
Subscore LOWER EXTREMITIES (0 – 16)	
**Total GMOS-R (0-38)**	

☐ **LMIC/UMIC**
☐ **HIC**

**Age-Specific Centile Rank within GM Category**	
